# Mathematical Modeling of Interleukin-35 Promoting Tumor Growth and Angiogenesis

**DOI:** 10.1371/journal.pone.0110126

**Published:** 2014-10-30

**Authors:** Kang-Ling Liao, Xue-Feng Bai, Avner Friedman

**Affiliations:** 1 Mathematical Biosciences Institute, The Ohio State University, Columbus, Ohio, United States of America; 2 Department of Pathology and Comprehensive Cancer Center, The Ohio State University, Columbus, Ohio, United States of America; 3 Department of Mathematics, The Ohio State University, Columbus, Ohio, United States of America; University of Torino, Italy

## Abstract

Interleukin-35 (IL-35), a cytokine from the Interleukin-12 cytokine family, has been considered as an anti-inflammatory cytokine which promotes tumor progression and tumor immune evasion. It has also been demonstrated that IL-35 is secreted by regulatory T cells. Recent mouse experiments have shown that IL-35 produced by cancer cells promotes tumor growth via enhancing myeloid cell accumulation and angiogenesis, and reducing the infiltration of activated CD8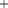 T cells into tumor microenvironment. In the present paper we develop a mathematical model based on these experimental results. We include in the model an anti-IL-35 drug as treatment. The extended model (with drug) is used to design protocols of anti-IL-35 injections for treatment of cancer. We find that with a fixed total amount of drug, continuous injection has better efficacy than intermittent injections in reducing the tumor load while the treatment is ongoing. We also find that the percentage of tumor reduction under anti-IL-35 treatment improves when the production of IL-35 by cancer is increased.

## Introduction

Interleukin-35 (IL-35) is a member of the IL-12 cytokine family. It is produced in human cancer tissues such as in melanoma, B cell lymphoma [1], lung cancer, colon cancer, esophageal carcinoma, hepatocellular carcinoma, cervical carcinoma, and colorectal cancer [2], [3], and it plays important roles in tumor progression and tumor immune evasion [1]. Fox3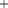 regulatory T cells (T_reg_) are common in tumor microenvironment [4], [5], where they induce immune-suppression. They do so by producing various cytokines, including TGF-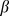, IL-10 [6], and IL-9 [7], thereby promoting tumor growth. It was also shown that T_reg_ secrete IL-35 [8]–[14]. IL-35 functions through IL-35R on various cell types, and is a potent immune-suppressor. Indeed, T_reg_-derived IL-35 was shown to inhibit antitumor T cell response [15], whereas IL-35-deficient T_reg_ have significantly reduced activity *in vitro* and *in vivo* [8]. Stable expression of EBI3, a gene that codes for IL-35 subunit, confers growth-promoting activity in lung cancer, whereas small interfering RNA silencing of EBI3 inhibits proliferation of lung cancer [16].

Recently Wang et al. [1] generated IL-35 producing plasmacytoma cancer cells and showed that the expression of IL-35 in tumor microenvironment increased the number of myeloid derived suppressor cells (MDSCs), and promoted tumor angiogenesis; furthermore, IL-35 inhibited the infiltration of cytotoxic T lymphocytes into the tumor microenvironment and rendered the cancer cells less susceptible to CTL destruction.

These experimental results suggest that blocking IL-35 may be an effective therapeutic approach to human cancer. To explore this possibility we develop in the present paper a mathematical model and then conduct *in silica* experiments to evaluate to what extend blocking IL-35 reduces tumor growth.

The model consists of a system of partial differential equations (PDEs) that involve interactions among cells (tumor cells, MDSCs, T cells, T_reg_s, endothelial cells) and cytokines (M-CSF, TGF-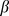, VEGF, IL-35). We first consider the situation which corresponds to the experiments in Wang et al. [1]. In these experiments two kinds of plasmacytoma cells were injected into wild type mice: tumor cells that have been transfected with IL-35 (J558-IL-35) so that tumor secretes high amount of IL-35 into the microenvironment, and “normal” plasmacytoma cells (J558-Ctrl) that secrete very small amount of IL-35. There is also a small amount of IL-35 produced by MDSC [17], [18] as well as IL-35 produced by T_reg_ [8]–[14]. We show that the model simulations agree with the experimental data in [1]. We also introduce, in this model, the effect of a drug which inhibits production of IL-35, and simulate various protocols for administering the drug. We find, that administering the drug frequently in small amounts yields better results than administering it infrequently in larger amounts. We also find that the percentage of tumor reduction under anti-IL-35 drug improves when the production of IL-35 by cancer is increased.

## Results

### Mathematical model

**Figure 1:**
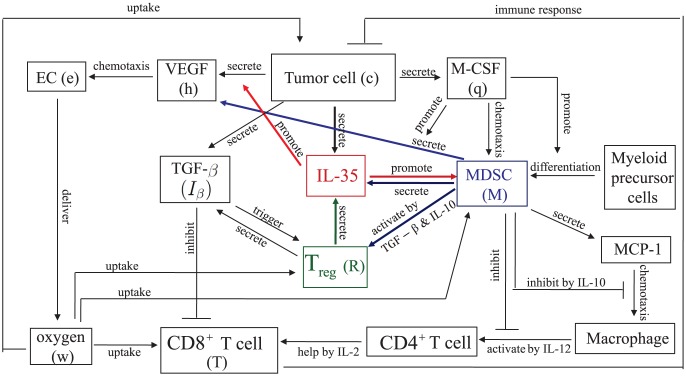
A network showing how IL-35 promotes tumor growth. M-CSF secreted by tumor cells promotes the differentiation of myeloid cells to MDSCs. M-CSF also attracts MDSCs to the tumor microenvironment by chemotaxis and promotes the secretion of VEGF by MDSCs. VEGF secreted by tumor cells and MDSCs attracts endothelial cells to trigger angiogenesis. IL-35 secreted by tumor cells, regulatory T cells and MDSCs promotes the secretion of VEGF by tumor cells and enhances the production of MDSCs. MDSCs promote T_reg_s, but also secrete MCP-1 to attract macrophages into the tumor microenvironment. Macrophages secrete IL-12 to activate CD4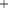 T cells, and CD4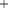 T cells secrete IL-2 which activates CD8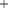 T cells. MDSCs also produce large amount of IL-10, which inhibits the chemotaxis and activation of CD4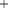 T cells.

The mathematical model is based on the network schematically shown in Figure 1. Cancer cells secrete M-CSF which attracts MDSCs; cancer cells and MDSCs secrete VEGF which triggers angiogenesis by attracting endothelial cells and enhancing their proliferation. The additional roles of MDSC are described in the caption of Figure 1. In particular, MDSC, inhibits the activation CD8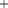 T cells via IL-10 and a variety of other mechanisms.

As mentioned in the Introduction, Wang et al. [1] considered two kinds of tumor cells injected into mice: J558-IL-35 and J558-Ctrl. In the case of J558-IL-35, IL-35 is produced mostly by tumor cells, less by T_reg_, and little by MDSC. In the case of J558-Ctrl, cancer cells produce very small amount of IL-35 so that IL-35 mainly comes from T_reg_ and MDSC. MDSC secretes TGF-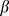 and IL-10 which promote T_reg_ [19], [20], and there is a positive feedback loop
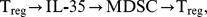
where the last activation is activated by TGF-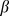 and IL-10.

We use the network described in Figure 1 to construct a system of partial differential equations. In order to simplify the computations we assume that the tumor and all the variables are radially symmetric. The variables of the model and their dimension are listed below.
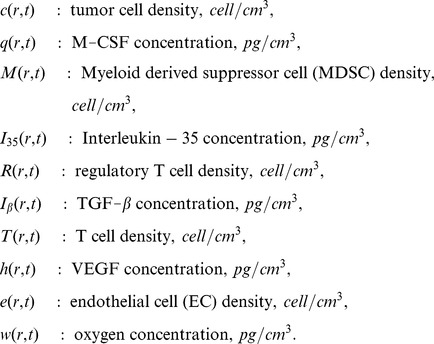



We proceed to write down the differential equation of each of the variables. Most of the parameters are taken from the literatures, as indicated; in Methods we explain how the remaining parameters were estimated.

#### Tumor cell (c)

The density 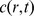 of tumor cells satisfies the following equation:
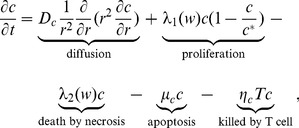
(1)where
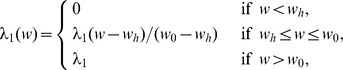





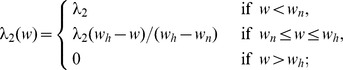




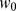
 is the oxygen level in heathy tissue, and the levels of oxygen for necrotic, extremely hypoxic, and intermediate hypoxic states vary in the intervals 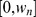, 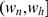 and 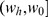, respectively.

The first term on the right-hand side of Equation (1) represents the dispersion (or diffusion) of tumor cells with diffusion coefficient 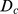. The second term accounts for the tumor proliferation, which depends on the concentration of oxygen 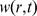 and tissue carrying capacity 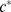. The third and fourth terms represent the death of tumor cells by necrosis and apoptosis, respectively. The last term accounts for the killing of tumor cells by activated CD8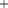 T cells [21]. The parameters in Equation (1) are listed in Table 1.

**Table 1:** Parameters for the tumor cell equation.

Parameter	Description	Dimensional	Reference
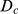	Diffusion coefficient of tumor cells	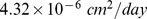	[22], [25] & estimated
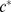	Carrying capacity of tumor cells	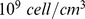	[22], [47], [55]
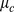	Apoptosis rate of tumor cell	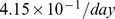	[22], [66]
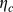	Killing rate of tumor cells from T cells	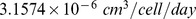	[55], [56] & estimated
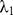	Maximal proliferation rate of tumor cells	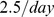	[22], [25], [67] & estimated
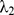	Maximal necrosis rate of tumor cells	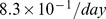	[22], [25], [55], [67]
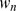	Lower bound of oxygen in necrotic	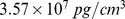	[22], [68]
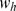	Lower bound of oxygen in extremely hypoxic	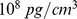	[22], [55], [68]
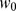	Normal oxygen level	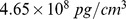	[22], [68]

#### M-CSF (q)

The concentration of M-CSF is given by the equation:
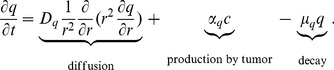
(2)


The first term on the right-hand side is the diffusion of M-CSF with coefficient 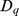. The second term represents the M-CSF secreted by tumor cells [19], [22], and the last term is the decay of M-CSF. The parameters in Equation (2) are listed in Table 2.

**Table 2:** Parameters for the M-CSF equation.

Parameter	Description	Dimensional	Reference
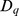	Diffusion coefficient of M-CSF	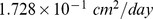	[22], [25], [55], [69], [70]
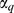	Production rate of M-CSF by tumor cell	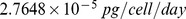	[22], [55], [71], [72]
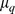	Decay rate of M-CSF	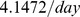	[22], [73]

#### Myeloid derived suppressor cell (MDSC) (M)

We model the dynamics of the density of MDSC by
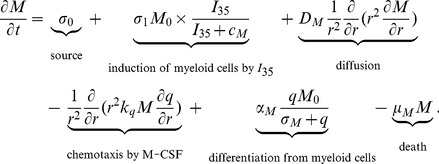
(3)


The first and last terms on the right-hand side account for the source and death of MDSCs. MDSCs undergo dispersion as well as chemotaxis driven by M-CSF (the third and fourth terms) [23]–[25]. It was reported in [1], that MDSCs do not undergo chemotaxis by IL-35 *in vitro* experiments. However, it has been observed that differentiation of MDSCs from myeloid precursor cells is enhanced by IL-35, although the mechanism is currently unknown [1]. We assume that this mechanism results in the second term on the right-hand side of Equation (3). The fifth term accounts for the differentiation of MDSCs from myeloid cells promoted by M-CSF [26]. The parameters in Equation (3) are listed in Table 3.

**Table 3:** Parameters for the MDSC equation.

Parameter	Description	Dimensional	Reference
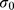	Source of MDSC	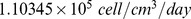	[56], [58] & estimated
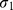	Maximal production rate via 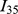	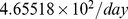	[1] & estimated
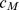		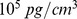	estimated
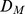	Diffusion coefficient of MDSC	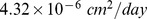	[22], [25] & estimated
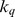	Chemotaxis rate of MDSC for M-CSF	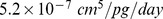	[25], [55]
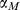	Polarization rate of MDSC by M-CSF	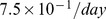	[56] & estimated
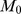	Density of myeloid precursor cells	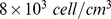	[56], [58]
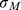		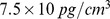	[56], [58]
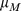	Death rate of MDSC	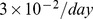	[58], [59]

#### IL-35 (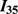)

The equation for the concentration of IL-35 is the following:
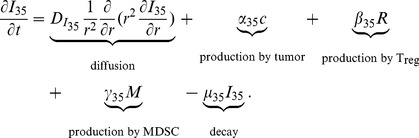
(4)


Experiments indicate that IL-35 can be produced by T_reg_s [8]–[14]. IL-35 possesses EBI3 and IL-12p35 subunits [1], [11], [13], [14], [27]. In human model, it has been shown that EBI3 was expressed in tumor infiltrating dendritic cells [17], [18], which is a subpopulation of MDSCs, and in lung cancer cells [2], [3], [16], whereas IL-12p35 was detected in EBI3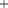 tumor cells [17], [18]. Hence, cancer cells and MDSCs could be other sources of IL-35 in human and mouse cancer. Accordingly, we include the production of IL-35 by cancer cells (the second term), T_reg_s (the third term), and MDSCs (the fourth term). For J558-IL-35 mouse model, we take 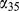 large enough and 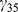 small enough such that, in our simulations, 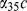 is relatively much larger than 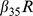, and 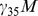 is significantly smaller than 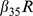. On the other hand, in the J558-Ctrl mouse model, we modify 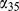 to be a much smaller than the value in J558-IL-35 case so that the production of IL-35 by tumor cells is significantly smaller than the productions of IL-35 by T_reg_s and MDSCs. The parameters in Equation (4) are listed in Table 4.

**Table 4:** Parameters for the IL-35 equation.

Parameter	Description	Dimensional	Reference
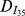	Diffusion coefficient of 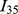	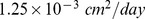	[60] & estimated
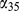	Production rate of 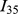 from tumor	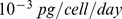 for J558-IL-35 mouse	[1], [16]–[18] & estimated
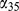	Production rate of 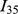 from tumor	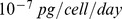 for J558-Ctrl mouse	[1] & estimated
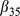	Production rate of 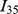 from T 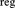	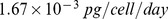	[34] & estimated
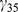	Production rate of 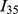 from MDSC	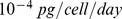	[17], [18] & estimated
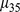	Decay rate of 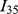	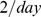	[61]–[63] & estimated

#### Regulatory T cell (R)

The equation for the density of regulatory T cells is given by
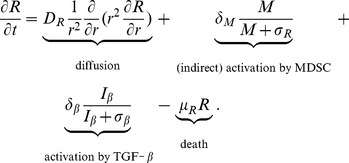
(5)


T_reg_ is activated by TGF-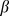 (the third term on the right-hand side) and by IL-10. IL-10 is secreted by MDSC [19], [20] and, for simplicity, we do not introduce IL-10 explicitly, and represent the activation of T_reg_ by IL-10 by the term 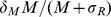. The parameters in Equation (5) are listed in Table 5.

**Table 5:** Parameters for the T_reg_ equation.

Parameter	Description	Dimensional	Reference
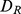	Diffusion coefficient of T_reg_	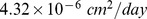	[22], [25] & estimated
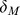	Maximal activation rate of T_reg_ by MDSC	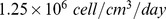	estimated
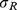		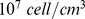	estimated
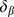	Maximal activation rate of T_reg_ by TGF- 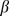	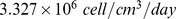	[38] & estimated
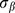		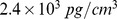	[38], [64] & estimated
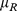	Death rate of T_reg_	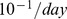	[34], [74], [75]

#### TGF-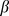 (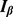)

The equation for the concentration of TGF-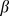 is the following:
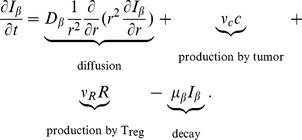
(6)


TGF-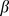 is secreted by tumor cells (second term) [28]–[35] and T_reg_s (third term) [36]–[38]. The parameters in Equation (6) are shown in Table 6.

**Table 6:** Parameters for the TGF-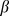 equation.

Parameter	Description	Dimensional	Reference
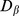	Diffusion coefficient of 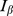	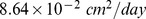	[76]
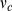	Production rate of 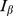 by tumor cells	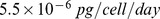	[34] & estimated
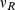	Production rate of 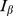 by T_reg_s	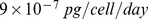	[34] & estimated
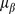	Decay rate of 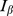	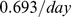	[76]

#### Activated CD8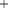 T cell (T)

Cytotoxic T cells (CTL), or CD8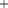 T cells, satisfy the equation:
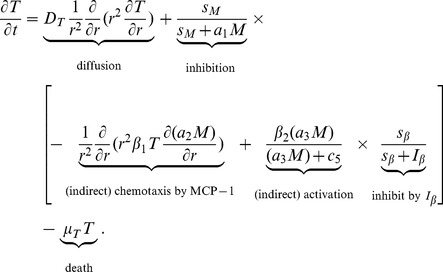
(7)


MDSC secretes MCP-1 which exerts chemotactic force on macrophages [39], [40], while macrophages secrete IL-12 which activates CD4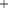 T cells [41] and CD4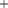 T cells produce IL-2 [42], [43] which activates CD8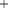 T cells. The activation of CD8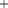 T cells is inhibited by TGF-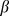 [44]–[46]. For simplicity we combine all these process by attributing the chemotactic force or CD8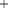 T cells and activation source of CD8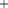 T cells to MDSC (the terms in square brackets in Equation (7)). The factor 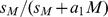 represents the fact that MDSC suppresses CD8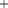 T cells proliferation by amino acid metabolism. The parameters in Equation (7) are listed in Table 7.

**Table 7:** Parameters for the CD8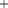 T equation.

Parameter	Description	Dimensional	Reference
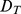	Diffusion coefficient of T cells	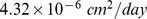	[22], [25] & estimated
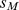		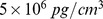	[58], [77] & estimated
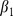	Chemotaxis rate of T cell from MCP-1	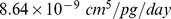	[78]–[80] & estimated
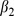	Activation rate from IL-12	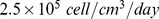	[58], [77] & estimated
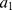	Production rate of IL-10 by MDSC	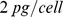	estimated
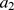	Chemotaxis rate of MCP-1 by MDSC	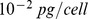	estimated
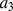	Production rate of IL-12 by MDSC	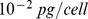	estimated
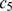		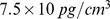	[56], [77] & estimated
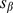		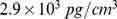	[34] & estimated
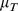	Death rate of T cells	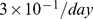	[58], [81]–[85]

#### VEGF (h)

The concentration of VEGF evolves according to the equation
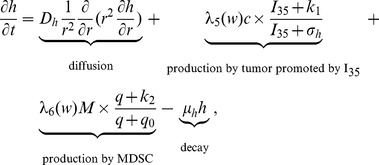
(8)where 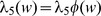 and 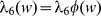 depend on the oxygen concentration 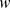, as follows:
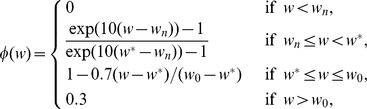
and 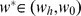 is the threshold at which the hypoxic effect on VEGF production by tumor cells and MDSCs is maximal. The function 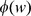 is chosen such that tumor cells and MDSCs can secrete VEGF under mild hypoxic conditions. The second term on the right-hand side of Equation (8) represents the VEGF produced by tumor cells and enhanced by 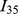 [1], and the third term accounts for VEGF produced by MDSCs and enhanced by M-CSF [47]; accordingly, the ratios 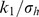 and 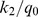 should be small. The parameters in Equation (8) are listed in Table 8.

**Table 8:** Parameters for the VEGF equation.

Parameter	Description	Dimensional	Reference
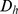	Diffusion coefficient of VEGF	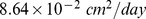	[22], [55], [86], [87]
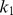		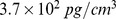	estimated
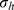	Critical value of 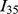	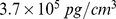	estimated
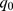	Critical value of M-CSF	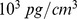	[22], [55]
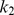		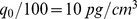	estimated
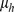	Decay rate of VEGF	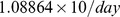	[22], [57]
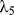		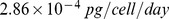	[22], [55] & estimated
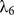		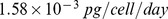	[22], [55]
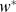		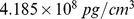	[22], [55] & estimated

#### Endothelial cell (EC) (e)

The equation of the density of EC includes dispersion, chemotaxis by VEGF, and proliferation by VEGF:
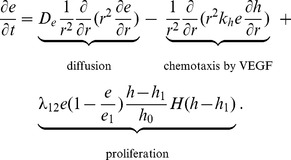
(9)


Here 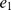 is the maximal density of EC inside the tumor, and 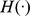 is defined by
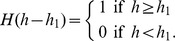



The last term, taken from [22], reflects the fact that VEGF induces proliferation of EC when the concentration of VEGF is higher than the threshold 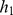. The parameters in Equation (9) are given in Table 9.

**Table 9:** Parameters for the EC equation.

Parameter	Description	Dimensional	Reference
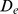	Diffusion coefficient of EC	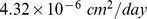	[22], [25], [57] & estimated
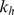	Chemotaxis force of EC by VEGF	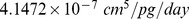	[22], [87] & estimated
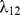	Proliferation rate by VEGF	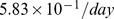	[88] & estimated
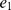	Maximal density of EC inside the tumor	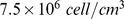	[22] & estimated
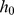	Scaling parameter for VEGF	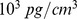	[89] & estimated
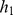	Threshold concentration of VEGF	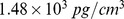	[90] & estimated

#### Oxygen (w)

We model the concentration of oxygen by the equation:
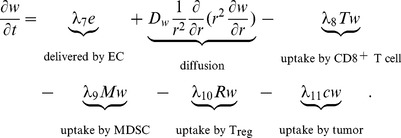
(10)


Oxygen is delivered by EC (the first term) and is taken up by CD8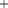 T cells (the third term), MDSCs (the fourth term), T_reg_s (the fifth term), and tumor cells (the last term). The parameters in Equation (10) are listed in Table 10.

**Table 10:** Parameters for the oxygen equation.

Parameter	Description	Dimensional	Reference
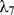	Delivery rate of oxygen	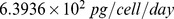	[55]
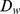	Diffusion coefficient of oxygen	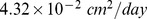	[25], [55], [69], [87]
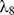	Consumption rate by T cells	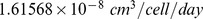	[55], [65] & estimated
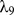	Consumption rate by MDSC	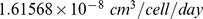	[55], [56], [65] & estimated
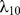	Consumption rate by T_reg_	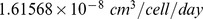	[55], [65] & estimated
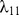	Consumption rate by tumor cells	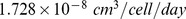	[55], [91], [92]

We assume that the tumor is radially symmetric and is contained in a sphere 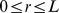, where 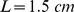.

We next introduce the initial and boundary conditions for each of the variables.

#### Initial conditions

We assume that the tumor cells are concentrated initially near 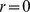, and take
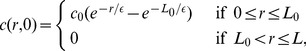
(11)with a positive parameter 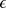, 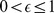, and scaling parameters 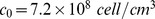 and 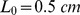. Since M-CSF is secreted by tumor cells, we take the initial concentration of M-CSF to be similar to the density of tumor cells,
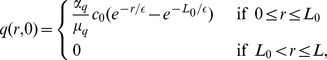
where the constant 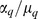 comes from the steady state equation for 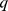.

Since tumor cells are concentrated at the center 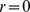, we assume that the MDSC is higher at the center and negligible near the boundary 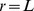,
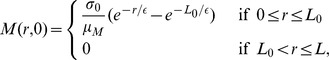
where the constant 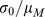 comes from the steady state equation of Equation (3). We assume that initially there are no activated CD8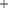 T cells, and take
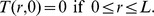



The activation of T_reg_s and the productions of 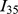 and VEGF are triggered by tumor cells and MDSCs; accordingly, we take
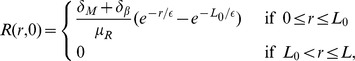





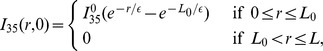





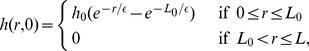
and 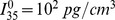, and 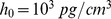. Similarly, 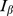 is produced by tumor cells and T_reg_s, so accordingly we take
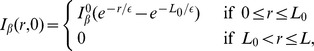
where 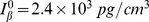.

Endothelial cells migrate into the tumor from the surrounding normal healthy tissue, so we take
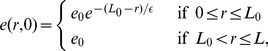
where 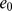 is the density of endothelial cell in normal healthy tissue. Finally, since endothelial cells represent capillaries through which oxygen is delivered, we prescribe
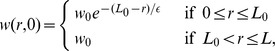
where 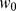 is the oxygen concentration in normal healthy tissue.

#### Boundary conditions

Since we assume radial symmetry, the first 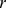-derivative of each variable vanishes at 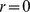. We assume no-flux condition at 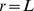 for all the variables except for the oxygen and endothelial cells, and we take
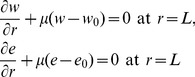
(12)where 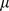 is the flux rate of EC from healthy normal tissue into the tumor microenvironment.

#### Parameters nondimensionalization

We nondimensionalizate the Equations (1)–(10) by the following scaling:
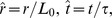





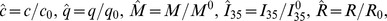





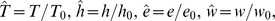





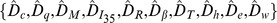





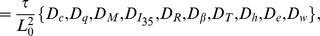





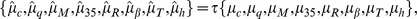





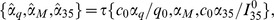





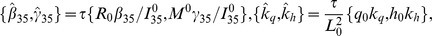





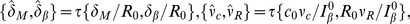





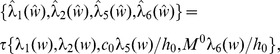





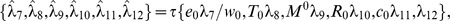





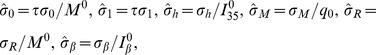





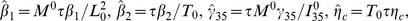





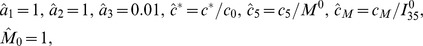





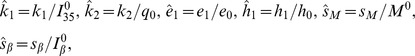
where the scaling parameters are
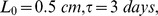





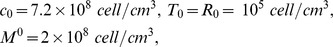





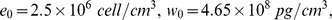





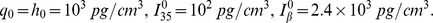



The dimensional and nondimensional values of all the parameters of Tables 1–10 are summarized in Tables 11 and 12.

**Table 11:** Model parameters and units.

Parameter	Dimensional	Dimensionless
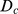	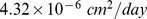	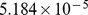
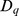	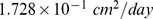	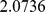
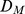	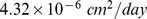	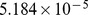
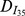	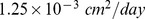	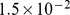
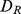	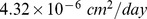	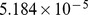
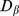	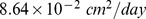	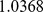
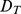	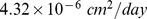	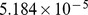
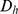	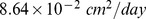	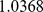
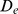	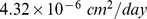	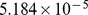
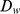	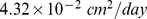	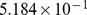
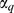	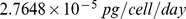	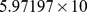
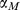	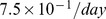	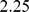
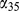	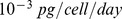 for J558-IL-35 mouse	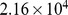 for J558-IL-35 mouse
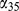	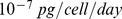 for J558-Ctrl mouse	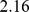 for J558-Ctrl mouse
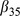	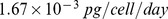	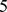
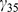	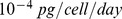	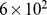
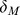	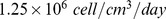	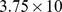
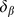	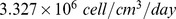	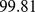
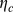	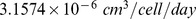	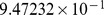
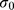	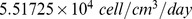	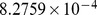
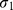	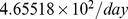	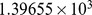
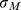	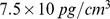	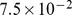
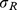	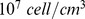	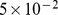
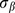	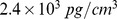	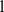
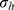	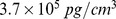	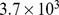
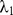	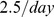	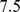
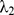	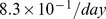	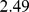
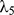	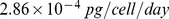	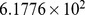
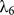	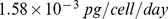	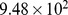
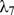	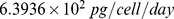	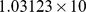
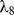	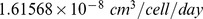	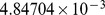
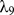	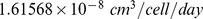	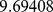
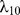	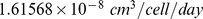	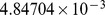
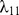	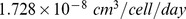	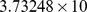
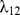	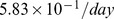	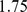
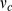	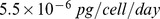	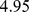
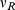	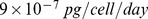	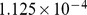
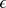	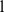	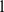
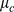	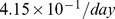	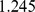
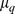	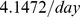	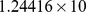
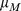	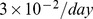	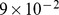
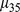	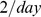	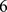
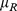	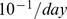	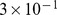
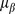	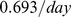	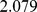
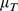	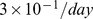	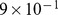
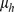	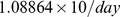	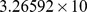
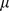	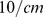	5

**Table 12:** Model parameters and units.

Parameter	Dimensional	Dimensionless
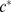	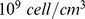	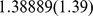
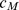	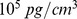	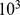
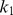	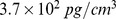	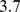
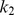	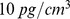	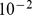
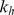	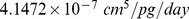	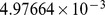
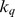	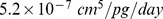	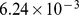
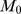	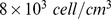	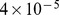
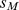	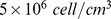	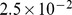
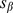	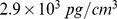	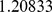
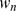	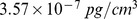	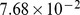
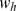	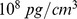	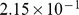
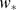	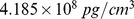	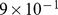
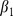	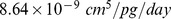	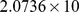
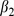	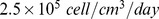	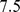
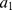	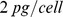	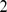
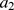	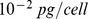	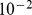
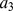	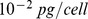	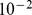
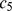	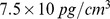	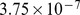
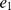	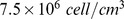	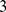
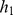	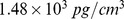	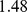
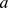	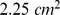	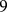
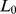	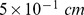	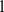
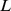	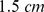	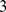
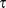	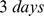	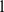
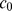	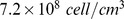	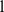
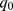	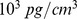	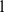
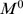	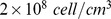	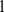
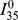	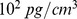	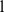
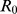	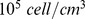	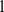
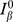	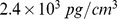	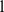
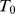	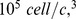	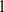
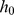	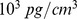	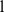
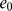	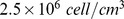	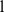
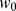	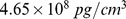	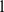

After dropping the symbol “
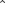
”, the model equations in the nondimensional form are as follows: 
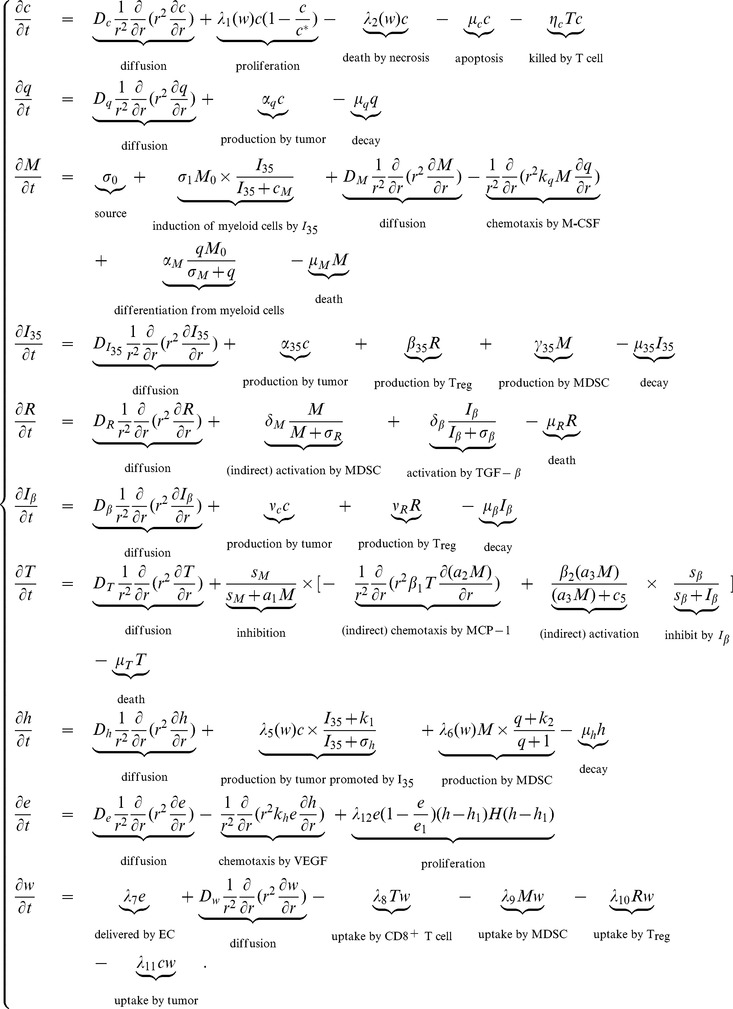
(13)


### Numerical simulation

In accordance with the experiments in Wang et al. [1], we consider two types of mice plasmacytoma J558 cells in wild type mice:

(i) J558-Ctrl tumor cells that secrete a very small amount of 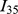.

(ii) J558-IL-35 tumor cells that secrete a large amount of 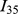.

**Figure 2:**
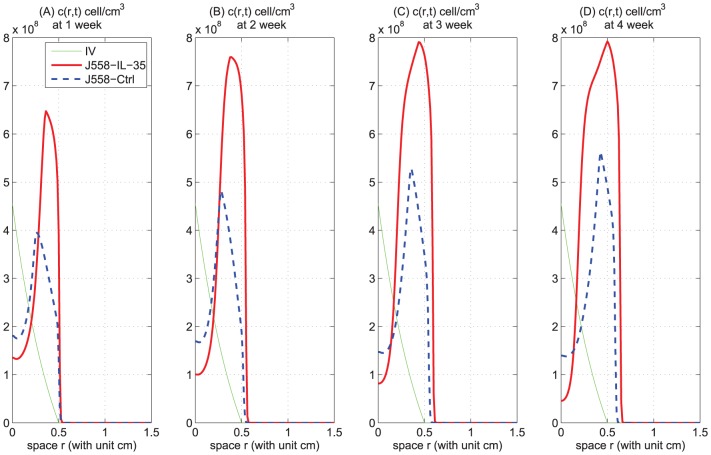
Spatial distributions of tumor cells. (A), (B), (C), and (D) are the spatial distributions of tumor cells 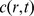 in the mice model at the end of the 2nd, 4th, 6th, and 8th weeks, respectively, for cases (i) and (ii). The thin curve is the initial value of tumor cells for the cases (i) and (ii). The solid curve is for J558-IL-35 tumor cells with large 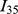 production (case (ii)) and the dashed curve is for J558-Ctrl tumor cells (case (i)).

We use matlab with 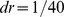 and 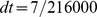 in nondimensional variables (i.e., 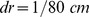 and 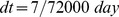 in dimensional variables). Figure 2 displays the spatial distributions of tumor cell density in cases (i)–(ii) at different times. We note that, in Figure 2, as time goes on, tumor cells migrate toward the boundary 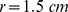, where oxygen is rich while tumor cell density is lower near the center 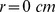, where oxygen is sparse. The migration speeds of these two cases (i)–(ii) are similar to each other, but tumor cells with larger 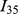 production (i.e., J558-IL-35 case) have higher peak during migration.

**Figure 3:**
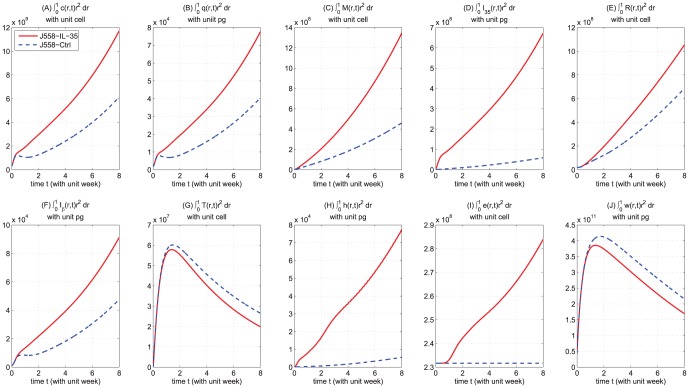
Evolution of cells and cytokines for J558-IL-35 and J558-Ctrl mice models. Panels (A) to (J) show the profiles of the total numbers of tumor cells, M-CSF, MDSCs, 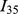, T_reg_s, TGF-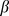, CD8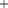 T cells, VEGF, endothelial cells, and oxygen, for cases (i) and (ii). The solid curve is for J558-IL-35 tumor cells with large 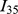 production (case (ii)) and the dashed curve is for J558-Ctrl tumor cells (case (i)).

The results of Wang et al. [1] were reported 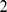 weeks after injection of tumor cells into mice. Hence, we compare our simulations at the end of the second week with the results in [1]. In Figure 3(C), the ratio for MDSC of J558-IL-35 to J558-Ctrl is 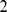, which is the same as Figure five A in [1]. In Figure 3(H), the ratio for VEGF of J558-IL-35 to J558-Ctrl is 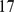, which is the approximately same as Figure four D in [1]. Next, we compare the ratio for T_reg_/CD8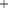 T cells of J558-IL-35 to J558-Ctrl with the result in [1]. But, in [1], they only showed the percentages of CD8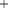/CD45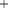, of CD4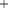/CD45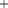, and of Foxp3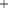/CD4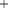. By combining these results (Figures seven B, seven D, and seven E in [1]), we find that this ratio (for T_reg_/CD8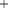 T cells) is 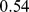. From our Figures 3(E) and 3(H), we compute the ratio of J558-IL-35 to J558-Ctrl to be 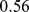. Thus in all the above three cases we get a very good quantitative fit with the experimental results of Wang et al. [1]. Finally, from Figure 3(A), we see that for tumor cells the ratio of J558-IL-35 to J558-Ctrl is 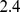, which is somewhat less than the ratio for the tumor volume of B16-IL-35 mice to B16-Ctrl mice in Figure three F in [1], and significantly less for J558-IL-35 mice. This discrepancy may be explained by the fact that *in vivo* the arrival of MDSCs to the tumor microenvironment is somewhat delayed and therefore the number of CD8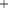 T cells in the control case is significantly less than in the J558-IL-35 case, while (for simplicity) our model does not include such a time delay.

The subunits of IL-35, EBI3 and IL-12p35, are highly expressed in cancers such as lung cancer, colorectal cancer, and esophageal carcinoma [2], [3]. Anti-IL-35 drug blocks the expression of IL-35 and could be an agent in treating these cancers [48]. To determine the effect of anti-IL-35 drug on cancer growth, we proceed to introduce it, as a drug, into our model. If we denote its concentration by 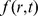 then all we need to do is to modify Equation (4) by
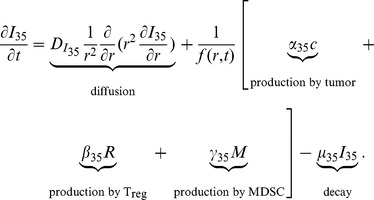
(14)


We make the pharmacokinetic assumption that 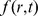 decreases in 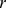 from the outer boundary of the tumor (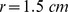) towards the center of the tumor (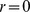), and take
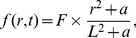
(15)where 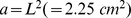 and 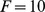. We shall compare several dosing schedules:

(i) no dosing of anti-IL-35, i.e., 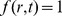, for all 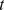 and 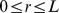;

(ii) continuous dosing with anti-IL-35 at fixed level 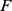 for 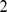 months,
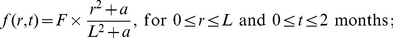
(16)


(iii) intermittent dosing for 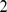 months, at double level 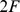, one week at a time with one week spacing between dosing,
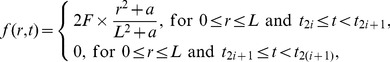
(17)for 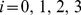, where 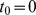 and the length of each interval 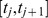 is one week.

**Figure 4:**
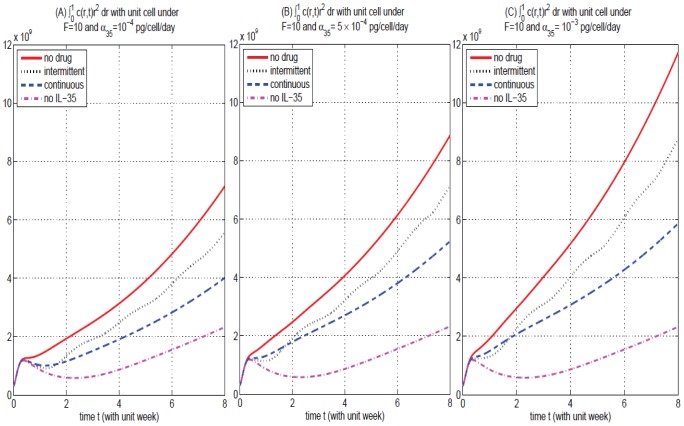
Comparison of continuous versus intermittent treatment in different production rate 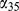 with drug strength 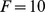. (A), (B), and (C) are the profiles of total numbers of 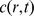, under 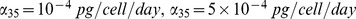, and 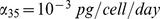, respectively. The solid curve is for case (i) that no dosing of anti-IL-35 in tumor cells. The dashed and dotted curves are for tumor cells with continuous (case (ii)) and intermittent (case (iii)) drug injections, respectively. The dashed-dot curve 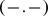 is the case that there is no IL-35 in the tumor microenvironment, i.e., 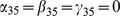 and 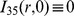, for 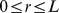.

We use matlab with 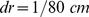 and 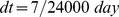 in dimensional variables. Figure 4 shows that the temporal growth of the total numbers of tumor cells, as functions of time, under
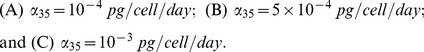



Figure 4 indicates that the continuous treatment has better efficacy in reducing tumor load than intermittent treatment when 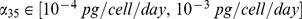. Figure 4 also shows that the reduction rate by anti-IL-35 is larger when tumor cells secrete higher amount of IL-35 as in Lung cancer and colorectal cancer [2], [3] than lower amount of IL-35 as in plasmacytoma [1]. Accordingly, as 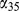 increases, the reduction in total tumor population becomes increasingly significant.

### Sensitivity analysis

In this section we perform sensitivity analysis on the parameters (in dimensional form) including those that were only roughly estimated and those that play important role in the model. We list these parameters with their ranges, baselines, and units in Table 13. We use the method described in Marino et al. [49], using the Latin hypercube sampling to generated 500 samples with 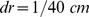 and 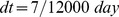.

**Table 13:** Parameters chosen for sensitivity analysis.

Parameter	Range	Baseline	Unit
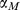	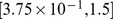	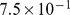	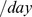
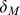	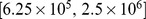	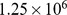	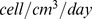
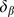	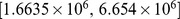	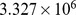	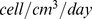
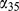	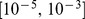	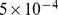	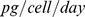
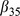	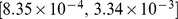	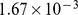	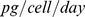
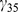	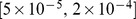	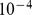	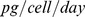
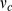	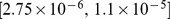	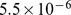	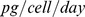
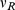	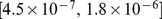	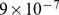	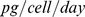
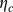	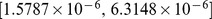	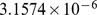	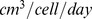
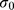	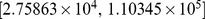	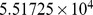	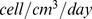
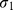	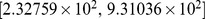	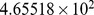	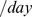
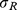	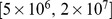	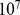	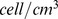
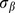	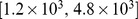	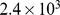	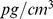
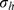	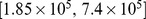	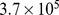	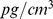
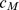	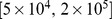	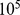	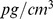
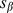	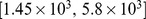	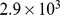	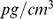
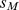	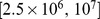	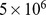	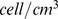
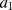	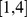	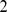	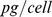
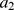	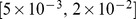	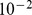	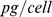
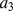	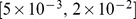	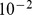	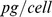
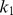	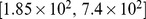	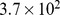	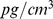
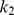	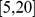	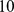	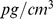
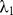	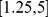	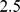	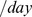
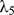	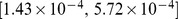	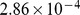	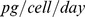
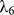	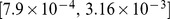	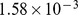	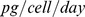
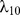	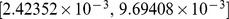	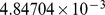	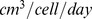
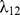	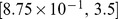	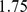	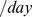
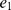	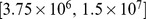	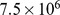	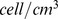
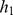	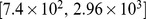	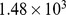	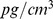

**Figure 5:**
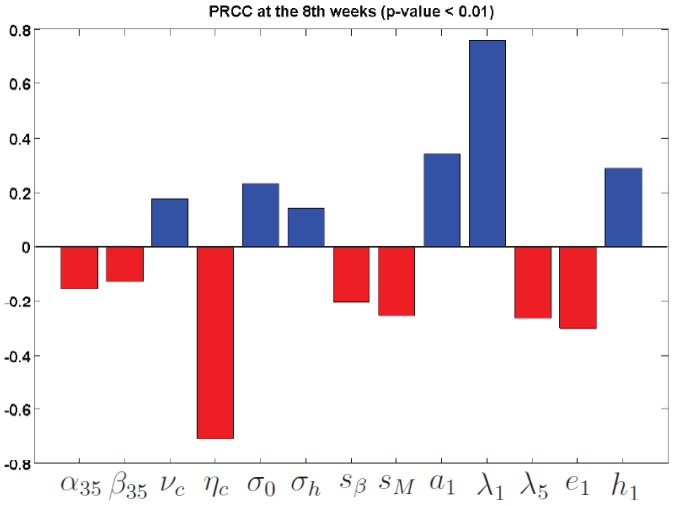
Sensitivity analysis. PRCC values at the second months for the parameters in Table 14 with p-value smaller than 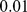.

Since we focus on how anti-IL-35 drug inhibits tumor growth, we calculate the partial rank correlation coefficients (PRCC) and p-value, corresponding to the ratio 
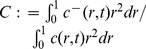
 for 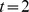 months, where 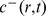 accounts for continuous treatment and 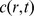 accounts for of no drug; 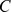 is a measure of the (relative) efficacy of the drug. In this analysis, all the parameters are chosen in the range from half to twofold of their baseline, except 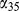 which is chosen from 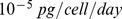 to 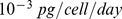. Table 14 lists the PRCC and their p-values. Figure 5 plots the PRCC of the parameters with p-values smaller than 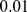. A negative PRCC (i.e. negative correlation) with p-value smaller than 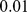 means that increasing this parameter value will decrease the value of 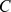 and hence increase the (relative) efficacy of the drug. A positive PRCC with p-value smaller than 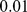 has the opposite meaning, that is, it will decrease the efficacy of the drug.

**Table 14:** The PRCC and p-value of parameters for sensitivity analysis.

Parameter	PRCC	p-value
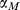	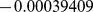	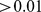
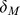	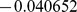	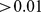
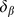	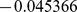	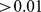
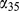	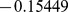	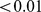
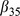	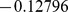	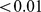
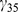	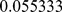	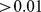
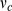	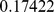	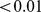
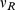	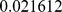	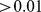
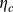	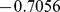	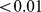
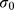	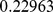	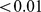
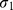	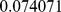	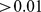
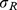	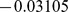	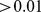
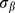	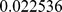	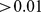
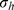	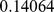	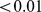
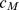	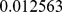	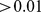
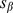	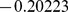	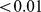
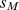	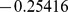	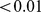
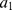	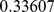	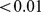
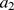	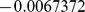	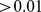
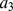	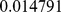	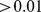
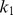	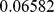	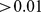
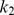	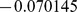	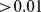
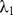	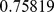	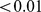
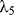	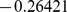	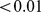
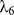	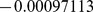	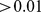
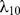	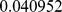	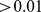
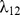	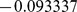	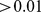
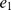	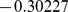	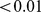
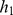	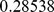	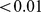

In Table 14, only 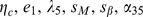, and 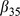 have negative PRCC with p-value smaller than 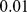. The most significant negatively correlated parameter is 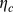. Larger 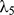 increases the production of VEGF and larger 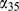 increases the production of 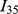 and both increase tumor load. The negative correlation of these parameters shows that the drug is more effective for tumor with higher rate of production of VEGF and IL-35. On the other hand, the negative correlation of 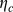 shows that the efficacy of the drug improves when the CD8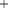 T cells are more affective in killing tumor cells. However, it is not true to conclude that, in general, the drug efficacy increases with larger tumor load, since larger 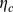 and 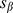 shrink the tumor load but yield better drug efficacy. Similar results hold for the parameters with positive PRCC. For example, larger 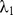 and 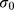 lead to higher tumor cell population while the tumor efficacy is decreased.

## Discussion

IL-35 is the most anti-inflammatory cytokine within the IL-12 cytokine family. In this paper we addressed the questions to what extend IL-35 is involved in tumor microenvironment and how effective is anti-IL-35 drug in reducing tumor growth. It is well known that T_reg_s are presented in the tumor microenvironment and that they secrete IL-35 to promote tumor growth. Recent mouse experiments of Wang et al. [1] determined the extend to which IL-35 enhanced the MDSC population and the VEGF concentration, and at the same time decreased the CD8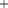 T cell population. Based on these experiments, we developed a mathematical model which includes in addition to tumor cells, MDSCs, CD8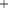 T cells, IL-35, and VEGF, also T_reg_s, endothelial cells, oxygen concentration, TGF-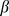, and M-CSF that is produced by cancer cells. The model is described by a system of partial differential equations. The simulations of the model are in qualitative agreement with the experimental results of Wang et al. [1].

We next extended the model to include anti-IL-35 as an anti-cancer drug. We compared the efficacy of the drug under two schedules: continuous versus intermittent injections of the same total amount of the drug. We found that continuous injection has better efficacy while the treatment is ongoing. Since it is well known that some cancers including lung and colorectal cancers most likely secrete large amounts of IL-35, we also investigated the efficacy of the drug for such cancers. We found that the percentage of tumor reduction under anti-IL-35 drug improves when the production of IL-35 by cancer is increased.

There are currently only few experimental results by which our model can be tested. In recent experiments by Nicholl et al. [50] it was demonstrated that IL-35 promotes pancreatic cancer cells proliferation while anti-IL-35 reduces this promotion. More specifically, in Figure three of Nicholl et al. [50] it is shown that IL-35 (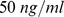) increases, on the average, by 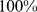 the proliferation of colonies of several pancreatic cancer cell lines, while in the presence of anti-IL-35 (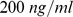) this increase is reduced to 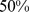. These *in vitro* results are in qualitative agreement with our results in Figure three (at week 8). Another example is taken from colorectal cancer in patients. As reported in Zeng et al. [2]. Foxp3
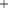
T_reg_ increases linearly with IL-35, and this is in qualitative agreement with Figures 3D and 3E of our simulations. As more experimental and clinical data become available, we should be able to test our model in more quantitative way, so that the model can further be refined.

In this paper we focused on the role of IL-35, although T_reg_ secrete besides IL-35 also other cytokines that promote tumor, such as IL-10 and IL-9 [7], [51]–[54]; these were not included directly in the present model, since we wanted to base the model on the recent experimental data by Wang et al. [1]. When data for other cytokines become available to the same precision as, for instance, in [1], our model could then be extended to include these cytokines, and to obtain a more comprehensive evaluation of anti-IL-35 efficacy in combination with other drugs.

## Methods

### Estimate 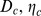 and 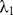 in Equation (1)

We assume that the killing efficiency of tumor cells by CD8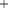 T cells is suppressed by IL-35 and that the proliferation rate of tumor cells is enhanced by IL-35. Accordingly in Equation (1), we choose smaller killing rate 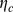 [55], [56] and larger proliferation rate 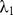 of tumor cells than in [22], [55]. For simplicity, we take all cells to have the same diffusion coefficient, 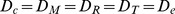, with 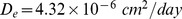 by [22], [25], [57].

### Estimate 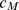 in Equation (3)

From Figures two B and three B in [1], we deduce that 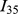 grows slowly in time, and
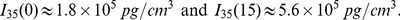
(18)


We take 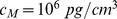 so that on the average 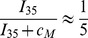, for 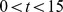 days.

### Estimate 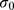, 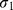, and 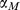 in Equation (3)

In order to estimate 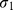, we use simplified forms of Equation (3):
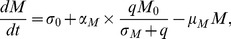
(19)




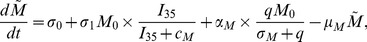
(20)for J558-Ctrl tumor cells and J558-IL-35 tumor cells, respectively. Taking the difference and recalling that on the average 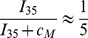 for 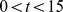, we get, with 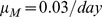 [58], [59],
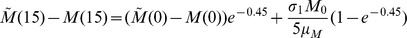
and the first term of the right-hand side may be neglected since initially the density of MDSC is small [1]. From Figure five A in [1], we deduce that
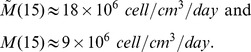
(21)


Since 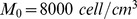 [56], [58], we get
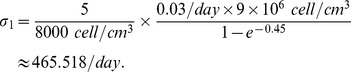



We assume that, due to the secretion of IL-35, the production of MDSC in the present model is larger than the production assumed in [56], so we have taken 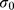 and 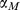 to be larger than in [56].

### Estimate 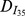 and 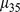 in Equation (4)

Since IL-35 belongs to the IL-12 family, we assume that its diffusion coefficient and its degradation rate are the same as for IL-12 [60]–[63]:
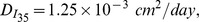





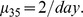



### Estimate 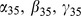 in Equation (4)

In order to find 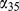 for the J558-IL-35 mouse model, we use the simplified version of Equation (4) where only cancer cells produce 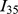, i.e., 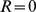 and 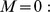


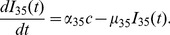
(22)


If 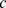 is taken to be a constant, then
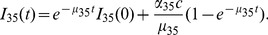
(23)


In the *in vivo* experiments of Wang et al. [1] the initial number of cancer cells that were injected was 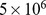 and we assume that they occupy a volume of 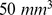, so that
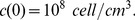
(24)


There is no data in [1] on the density of the tumor cells in day 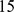, but the tumor cells were observed to grow rapidly in the first 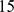 days. We assume that the average of the density of tumor cells in the first 15 days is very close to the maximal capacity 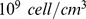 and take, in (23), 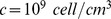 for J558-IL-35 tumor cells. Recalling Equation (18), we get, with 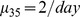 (Table 4),
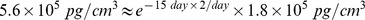





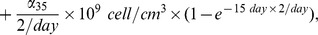
so that 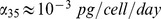 for J558-IL-35 mouse model.

In contrast to the case of J558-IL-35 mouse model, in J558-Ctrl mouse 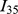 is mainly secreted by T_reg_s [11], [13], [14], [27], little by MDSCs, and very little by tumor cells. Hence, in the J558-Ctrl case, we take the production rate of 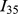 by tumor cells to be 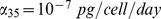.

The production rate of 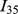 by T_reg_ is estimated to be 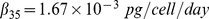 [34] and we take the production rate of 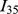 by MDSCs to be small enough, i.e., 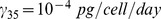, so that the production of 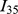 in the J558-IL-35 case satisfies:
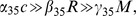
and production of 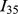 in J558-Ctrl case satisfies:
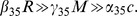



### Estimate 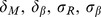 in Equation (5)

In [38], the cytokine signalling by TGF-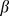 on T_reg_ is modeled by
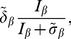
(25)where 
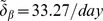
 which has dimension per day and 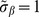 which is nondimension. In our Equation (5), the dimension of 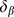 is 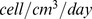 and the dimension of 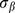 is 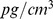. Correspondingly, we take
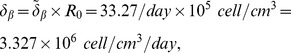


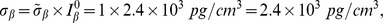
where 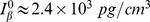 [64].

MDSC also activates T_reg_ population. We assume that the activation of T_reg_ by MDSC is weaker than the activation of T_reg_ by TGF-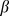, and hence take it to be
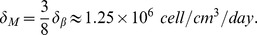



We also take 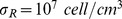.

### Estimate 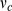 and 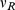 in Equation (6)

We assume as before that the initial tumor occupies a volume of 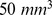 and, accordingly, also T_reg_ occupies the same volume. In [34], the production of 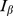 by tumor cells and T_reg_s are 
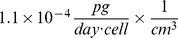
 and 
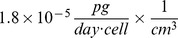
, respectively. Hence,
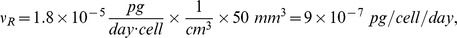





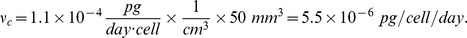



### Estimate 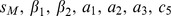 in Equation (7)

Since IL-35 enhances the population of MDSC, the concentration of IL-10, which we represent by 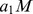, is larger than the one in [56]. Hence, we chose 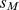 to be larger than the corresponding value of 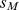 in [56]. Moreover, since IL-35 promotes tumor growth, we expect a stronger immune response by T cells than in [56] and hence we take 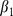 and 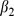 larger than the corresponding value in [56]. The parameter 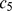 is taken from [56]. Since the chemotaxis and activation of CD8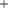 T cells are indirect, we take 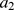 and 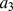 to be smaller than 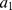: 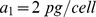 and 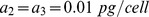.

### Estimate 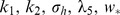 in Equation (8)

We take 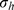 to be the average of the concentration of IL-35 at times 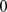 and 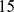 days, so that 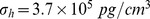 by Equation (18). We assume that the productions of VEGF by tumor cells and MDSCs are small when there are no IL-35 and M-CSF, respectively, so we set 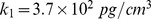 and 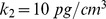. Since in [1] 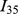 increases the concentration of VEGF significantly, we take 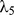 to be larger than the value in [56]. We also slightly modify the parameter value 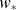 and function 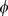 used in [56].

### Estimate 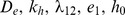, and 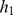 in Equation (9)

We take values similar to those in [22], [55].

### Estimate 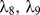, and 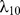 in Equation (10)

We assume that CD8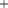 T cells, MDSCs, and T_reg_s have the same consumption rates of oxygen, so we take 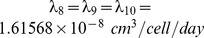 [55], [56], [65].
